# Stochastic adaptation and fold-change detection: from single-cell to population behavior

**DOI:** 10.1186/1752-0509-5-22

**Published:** 2011-02-03

**Authors:** Tatiana T Marquez-Lago, André Leier

**Affiliations:** 1 Department of Biosystems Science and Engineering, ETH Zurich, Universitätsstrasse 6, CH-8092 Zurich, Switzerland

## Abstract

**Background:**

In cell signaling terminology, adaptation refers to a system's capability of returning to its equilibrium upon a transient response. To achieve this, a network has to be both sensitive and precise. Namely, the system must display a significant output response upon stimulation, and later on return to pre-stimulation levels. If the system settles at the exact same equilibrium, adaptation is said to be 'perfect'. Examples of adaptation mechanisms include temperature regulation, calcium regulation and bacterial chemotaxis.

**Results:**

We present models of the simplest adaptation architecture, a two-state protein system, in a stochastic setting. Furthermore, we consider differences between individual and collective adaptive behavior, and show how our system displays fold-change detection properties. Our analysis and simulations highlight why adaptation needs to be understood in terms of probability, and not in strict numbers of molecules. Most importantly, selection of appropriate parameters in this simple linear setting may yield populations of cells displaying adaptation, while single cells do not.

**Conclusions:**

Single cell behavior cannot be inferred from population measurements and, sometimes, collective behavior cannot be determined from the individuals. By consequence, adaptation can many times be considered a purely emergent property of the collective system. This is a clear example where biological ergodicity cannot be assumed, just as is also the case when cell replication rates are not homogeneous, or depend on the cell state. Our analysis shows, for the first time, how ergodicity cannot be taken for granted in simple linear examples either. The latter holds even when cells are considered isolated and devoid of replication capabilities (cell-cycle arrested). We also show how a simple linear adaptation scheme displays fold-change detection properties, and how rupture of ergodicity prevails in scenarios where transitions between protein states are mediated by other molecular species in the system, such as phosphatases and kinases.

## Background

Chemical reactions inside cells have long been correctly described as both discrete and stochastic [1-3], often entailing acute spatial patterns or dependencies [4-6]. Despite the intrinsic uncertainty in the occurrence of these chemical events, and basically against all odds, cells prevail as efficient decision makers. Not only are their fate decisions influenced by stochastic events and embedded within widely fluctuating environments, but they are stochastic themselves [7], the underlying mechanisms of which remain widely unknown.

So, one cannot help but wonder: how do cells process widely varying information from their environment, control their own chemical 'noise', and still manage to produce appropriate responses? The key to this question lies in signal transduction pathways, a series of interconnected chemical events that lead to highly specific cell responses. One such mechanism is adaptation, a common term used to represent sets of chemical reactions that generate a transient response in the presence of a sustained stimulus [8]. These transient responses have been shown to affect gene expression and regulatory processes, where the cell decision is determined by the strength and duration of the input signal [9].

Adaptive behavior can result from three basic signaling motifs: integral control, negative feedback, and feed-forward regulation [8]. The first is an abstraction of an engineering principle, where regulation is achieved by integrating the differences between a desired response and the state of the system. A cellular system may proceed in a similar fashion, by comparing 'actual' to 'desired' conditions, as has been found to be the case in bacterial chemotaxis [10-13] or calcium homeostasis [14].

Integral control can be achieved through appropriate combination of negative feedback loops, the latter of which are ubiquitous elements of signaling pathways, allowing for myriads of types of physiological homeostasis. In a self-regulating gene, a transcriptional repressor negatively regulates its own expression and, within certain network architectures and ranges of feedback strength, noise can be effectively reduced. In this sense, negative feedback allows a system to respond by decreasing the magnitude of any input perturbation, generally resulting in stabilization of the input signal. However, while the latter is true in a deterministic setting, several types of non-classic behavior can be observed once considering discrete signals and stochasticity [15].

In contrast, feed-forward architectures let the system respond to known cues (input signals) in a predetermined way, independently of the system's response. This is the essential difference from feedback mechanisms, where the output influences ('feeds back') the system to create a new response. For feed-forward to produce adaptation, two signal-dependent pathways must affect a third component, in opposite ways, otherwise known as 'incoherent' feed-forward loops [16].

Several exhaustive studies have shown that negative feedback regulation rarely yields perfect adaptation, whereas integral control and feed-forward regularly do so [8,10,11,17]. Nevertheless, it should be noted that negative feedback can produce adaptation states close to 'perfect', and basically indistinguishable in terms of biological functions [8]. By perfect adaptation it is generally understood that the system will return to the exact state where it was before the input signal was introduced, provided the system was already in equilibrium.

Furthermore, in order to consider a system adaptive, certain eligibility criteria in terms of amplitude and duration of the system response have to be met. It should be noted that no homogeneous criteria exist in the literature, and comparison between different adaptation models can become a daunting task. Quite generally, though, amplitude has been assessed in terms of sensitivity and precision, namely, the difference between maximal response and pre-stimulation values, and the difference between equilibrium values before and after stimulation, respectively [18,19].

Recently, some types of adaptive systems (such as the incoherent feed-forward loop) have been shown to display fold-change detection (FCD) properties. Namely, that the system generates a response to fold-changes in the input signal, rather than absolute levels [20,21]. The latter is related to Weber's law, which describes the relationship between a stimulus and its perceived intensity, a widely used concept in perception studies.

In this respect, some experimental studies have shown how important transduction mechanisms (such as ERK2 translocation [22] or Wnt signaling [23]) display robust fold-change responses. From these studies, several hypotheses have already arisen, such as whether cells detect and process information in relative rather than absolute terms, or whether fold-change detection facilitates the production of adjustable noise filters. Proving such hypotheses would greatly aid our understanding of cell signaling pathways, as FCD could rescale meaningful signal changes with respect to the background noise.

With all these points in mind, and in response to some of the open questions posed in [18], we study the effects of stochasticity in a minimalistic adaptation architecture, a 'two-state protein' scheme [24,25]. For such, we wanted to analyze how stochastic profiles in a single-cell system propagate to population behavior, and what this actually entails in terms of system predictability. Surprisingly, our preliminary simulations highlighted how single cell and population behavior can be completely different, adaptation largely being an emergent property of a large ensemble. This led us to analyze adaptation in an exact stochastic setting, and understand why one should think of adaptation processes in probability space, rather than in numbers of molecules.

Until now, no one had noticed how ergodicity breaks down in simple linear scenarios devoid of cell growth and replication properties. Hence, our results provide key novel insights that need to be considered in any future study of adaptation, as well as any study where biological ergodicity is readily assumed. An example of the latter is linear and nonlinear signaling pathway studies.

Additionally, we also respond to some of the open questions in [21] and show how the simple linear 'two-state protein' scheme in a stochastic setting displays fold-change detection properties, both for consecutive stimulation inputs and separate fold-stimulations. This is the first study of FCD under stochasticity, the importance of which extrapolates to any cell signaling study.

Lastly, we discuss how extensions of the 'two-state protein' scheme (by considering discrete mediators, e.g. kinases and phosphatases) retain many of the properties observed in the purely linear system, including rupture of ergodicity.

## Results

**Figure 1:**
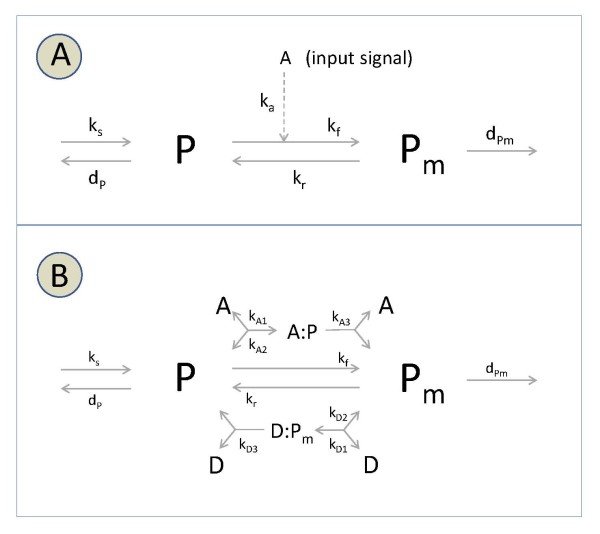
**Two-state protein models**. (a) Basic linear scheme, and (b) extension to account for kinases and phosphatases mediating changes between states.

In what follows we shall consider the simplest adaptive architecture, previously described in the literature as a single 'two-state protein' scheme [24,25]. The idea behind this model is to consider a protein in an unmodified and modified state, denoted as *P *and *P_m_*, respectively. The switch between the two states of the protein has basal rates *k_f _*and *k_r_*, and is additionally influenced by an input signal A with rate *k_a_*. Moreover, the total protein concentration is allowed to vary in time, and neither the synthesis of the unmodified protein (*k_s_*), nor the distinct degradation of the two states of the protein (*d_P _*and $d_{P_{m}}$), depend directly on the input signal (Figure 1A).

The analysis in [25] highlights several key issues. First, the steady state values of the protein states are proportional to the input signal, one being inversely proportional, the other being directly proportional. Second, when either protein degradation rate is close to zero (or much smaller than the other), the steady state concentration of the other protein is (nearly) independent of the signal. Such independence hints at the potential adaptation to changes in the input signal, a feature that can be readily observed when considering several parameter combinations. Furthermore, the choice of parameters will determine the sensitivity with respect to repeated stimulation. Namely, whether the system responds and adapts to consecutive input signals, or not.

The key questions here are: (1) which of the above mentioned properties hold when discreteness and stochasticity are taken into account, (2) do these properties only hold at the single-cell level, or extend to multi-cell schemes implying a kind of biological ergodicity, and lastly (3) does this system display fold-change detection properties?

### Adaptation in a stochastic setting

The chemical master equation (CME) describes the time evolution of the probability *P*(*X*, *t*), for having ***x ***= [*x*_1_,...*x_N_*] molecules at time *t *in a system with *R *elementary reactions, *N *molecular species, and volume Ω. Namely, it describes the evolution of all possible states of a chemical system in probability space.

If we consider the 'two-state protein' scheme (Figure 1A) in a stochastic setting, its time evolution will be described by the following CME:

$$\begin{array}{c} \frac{\partial P_{n,m}}{\partial t}=\;\;k_{s}\left(P_{n-1,m}-P_{n,m}\right)\\ +\;d_{P}\;\left(\left(n+1\right)P_{n+1,m}-n\;P_{n,m}\right)\\ +\;k_{f}\;\left(\left(n+1\right)P_{n+1,m-1}-n\;P_{n,m}\right)\\ +\;k_{a}A\;\;\left(\left(n+1\right)P_{n+1,m-1}-nP_{n,m}\right)\\ +\;k_{r}\;\left(\left(m+1\right)P_{n-1,m+1}-mP_{n,m}\right)\\ +\;d_{P_{m}}\;\left(\left(m+1\right)P_{n,m+1}-mP_{n,m}\right) \end{array}$$

where the index *n *(*m*) in *P_n.m _*denotes the number of proteins in state *P *(*P_m_*). Additionally, we prescribe an input signal following the step function *A*= *S*·*F_i_*, where *S *is in units of *μM*

$$S\;=\;\left\{ \begin{array}{l} 0\;\text{if}\;t\in [0,50)\;\;\text{or}\;t\in \;[100,150)\\ 1\;\text{if}\;t\in [50,100)\;\;\;\text{or}\;t\in \;[150,200)\\ 2\;\text{if}\;t\in [200,250)\\ 3\;\text{if}\;t\in [250,300)\\ 4\;\text{if}\;t\in [300,350) \end{array}\right.$$

the factor *F_i _*is the i^th ^signal scale parameter of *F *= (0.01,0.1,1,10,100), and the macroscopic reaction rate constants are set as follows: *k_s _*= 0.01, *k_a _*= 1, *k_f _*= 1, *k_r _*= 10, *d_P _*= 0.01, $d_{P_{m}}\;=\;1$. As is usual in a stochastic analysis, concentrations and 0^th^/2^nd ^order reaction rates have to be scaled by the factor *V*·*A_v_*, where *V *is the volume and *A_v _*denotes Avogadro's constant (approximately 6.02214179 × 10^23 ^molecules^-1^). Here, we consider a volume of 1 femtoliter and selected initial values close to equilibrium. Namely *P *= 0.1 and *P_m _*= 0.01 *μM*, corresponding to 60 and 6 molecules, respectively.

Now, stochastic processes can be studied by trajectory based approaches or by obtaining their underlying probability distribution function (PDF), which tracks how the probability of having specific numbers of molecules in the system changes over time. This is a daunting - many times unfeasible - task, given the combinatorial explosion of the number of coupled differential equations to consider, corresponding to increasing numbers of possible states of the system.

In fact, whenever a system is solely composed of 0^th^/1^st ^order reactions, exact analytical PDF solutions can be obtained [26,27]. Thus, exact analytical expressions can be derived for the first two moments, which have been shown to match the solution of the system translated to a stochastic differential equation (SDE) problem [28]. Moreover, in such linear cases the first moment of the SDE solution will converge to the ODE solution, a consequence of the linearity in the drift term.

**Figure 2:**
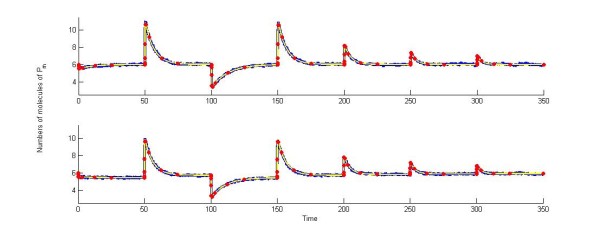
**Solutions match when using different modeling regimes (*F_i _*= 1)**. Mean of 10,000 simulations using (A) *d_P _*= 0, yielding perfect adaptation, and (B) *d_P _*= 0.01, corresponding to near-perfect adaptation. Trajectories portray SSA simulations (blue), the first moment of exact CME solution using finite state projections (red), and solution of corresponding ODE system (yellow). Input signal 'stimulation' intervals were spaced by 50 units of time between one another, while each subinterval was subdivided into the following time points: [50/2^14 ^50/2^12 ^50/2^10 ^50/2^8 ^50/2^6 ^50/2^4 ^50/2^2 ^25 50]. We restricted our state space to all combinations of (*P, P_m_*) such that *P *∈ [0,110] and *P_m _*∈ [0,30], guaranteeing our obtained PDF is closer than 99.99% to the exact solution of the CME, at all considered time points.

**Figure 3:**
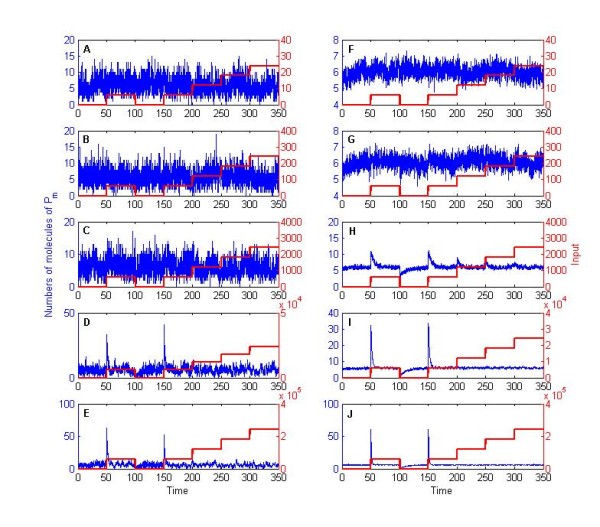
**Stochastic trajectories for chemical species P_m _in the basic two-state protein model**. (A-E) Single and (F-J) mean over a population of 50 cells. Subfigures correspond to different signal tuning parameters: (A, F) *F_i _*= 0.01, (B, G) *F_i _*= 0.1, (C, H) *F_i _*= 1, (D, I) *F_i _*= 10, and (E, J) *F_i _*= 100. Trajectories portray SSA simulations (blue) and input signal in numbers of molecules (red).

Hence, in our case, if we compare the mean of 10,000 stochastic trajectories, the corresponding deterministic solution, and the expectation of the CME reported as the sum over equal numbers of molecules of *P_m _*in the exact PDF solution, it comes as no surprise that all solutions nicely match (Figure 2). As had been previously reported, the time evolution of *P_m _*can show near-perfect adaptation when $d_{P}\ll d_{P_{m}}$, while *d_P _*= 0 yields perfect adaptation. However, and quite intriguingly, if we focus on individual SSA trajectories, no adaptive behavior can be inferred from single trajectories for certain values of the input signal (e.g. Figure 3C, corresponding to *F_i _*= 1). The key behind this issue lies in what the solution of the CME really entails: a set of time dependent values in probability space. In this sense, computing the expected value masks how often discrepancies from this mean can happen, as well as their potential magnitude, possibly leading to entirely different dynamics. Hence, a more appropriate description of the system lies in describing the evolution of the probability, and not the expectation, in time.

To analyze this further, let us focus on perfect adaptation systems considering *F_i _*= 1, *P *∈ [0,110] and *P_m _*∈ [0,30], for which we will obtain exact PDF solutions of the CME by using finite state projections [29]. This implies the solution of the CME will be both exact and complete whenever these ranges cover all possible reachable states, portraying the full probability space instead of solely describing single exact trajectories.

**Figure 4:**
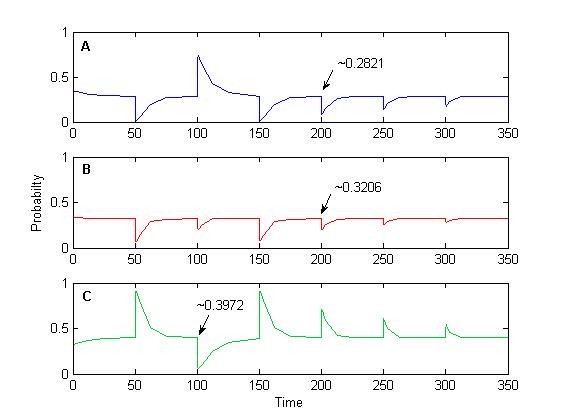
**Evolution of the probability using finite state projections, for separate sets of numbers of molecules of *P_m_***. Here, we consider the perfect adaptation system (*d_P _*= 0) with *F_i _*= 1, and distinguish between: (A) 0 to 4, (B) 5 to 6, and (C) 7 to 30 molecules of *P_m_*.

One can notice the deterministic adaptation value in our 'two-state' protein scheme lies close to 6 molecules of *P_m_*. If we now track the evolution of the probability (i.e. the exact solution of the CME) in three separate sets: 0 to 4, 5 to 6, and 7 to 30 molecules of *P_m _*it can be observed there is indeed sensitivity to the input signal and relaxation to pre-stimulus values, albeit in a probabilistic context (Figure 4). To understand what this entails, let us first focus on the system at time *t *= 50 Here, the probability of all states containing 0-6 molecules of *P_m _*will decrease once the signal is introduced (Figure 4A), as would be expected by the sudden shift of *P_m _*to higher values in the deterministic setting. In contrast, as we approach *t *= 100, the probability of all states containing 5-30 molecules of *P_m _*decreases (Figure 4C). However, one should notice that, at *t *= 100 (and any other end of each stimulation interval), the system will revolve around states with 5 to 6 molecules of *P_m _*with a probability of occurrence of roughly 32% (Figure 4B). This leaves 'a lot of room' (the remaining ~68%) for the system to be located elsewhere, as can be readily observed from single cell simulations (e.g. Figure 3C). Furthermore, of these 32%, only half belong to having 6 molecules of *P_m _*in the system, which is closest to the deterministic solution. Most importantly, differences in equilibrium values entail wide variations for initial conditions of the next simulation interval, yielding the observed heterogeneity in single cell behavior.

The description above highlights a property of any stochastic description: a system cannot be guaranteed to be in any state, and probabilistic bounds are the maximal level of information. However, one should notice the signal in this example is at least 2 orders of magnitude larger than any other component of the system. Hence, the 'two-state protein' scheme provides for a counter-intuitive example where local noise overrides the effect of a strong external signal, and adaptation behavior can become an emergent property only attained at the (mean) population level. Moreover, as could be expected from the deterministic solution, the probability profile also portrays loss of sensitivity with repeated stimulation.

### **P**opulation behavior

Ensemble measurements (such as those obtained from flow cytometry) display cell characteristics as distributions of values calculated over large samples of cells and, many times, distributions of cells at certain stationary states are used to infer the stochastic behavior of a single cell. By doing so, a biological version of ergodicity is necessarily implied: the percentage of the cell population in a particular state is identical to the probability to find a single cell in that state [30].

Mathematically, the ergodicity of a stochastic process is guaranteed if (i) the stochastic process is a finite-state Markov process and (ii) the stochastic process converges to a stationary state for any initial condition [1]. However, such assumptions have many times been relaxed or misinterpreted and, in such cases, erroneous conclusions with respect to single cell behavior could have been derived from population data [30].

Recently, many studies in the literature have addressed this issue. Population distributions have been based on chemical Langevin equations [3,31,32], and clever solution methods have been developed by noticing a Sturm-Liouville operator. For instance, the authors in [31] highlight the potential bias of population measures when cell replication rates are not homogeneous, or depend on the cell state. Similarly, the authors in [30] support the latter findings and further discuss how ergodicity breaks down whenever there are chemical interactions between cells, and when single cells display 'complicated' behavior (such as bistability).

Our exact solution of the two- state protein system shows how ergodicity cannot even be assumed in minimalistic linear examples. Our linear system underlies a finite-state Markov process, is expected to relax to a stationary state, and is devoid of possible effects via cell replication and chemical interactions between cells. However, for certain input signal strengths, biological ergodicity still breaks down. In a nutshell, when the signal tuning parameter is set as *F_i _*< 1, no adaptive behavior is observed in either single cells or populations. In contrast, when considering *F_i _*> 1, both single cells and populations exhibit adaptation. However, when *F_i _*= 1, single cells need not display any apparent sensitivity to the input signal, while both sensitivity and adaptive behavior can be observed at the population level (Figure 3C and 3H).

**Figure 5:**
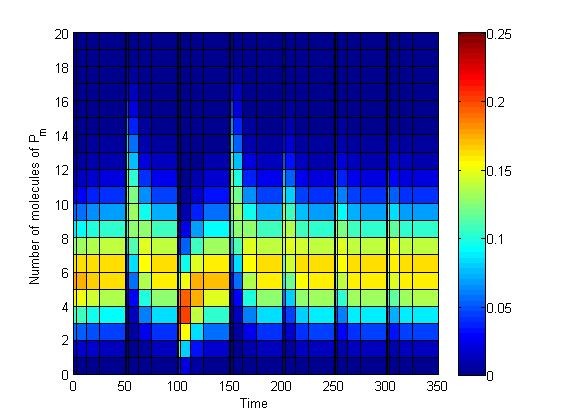
**Heat map of CME solution in PDF form, perfect adaptation case (*d_P _*= 0)**. Input signal 'stimulation' intervals were spaced by 50 units of time between one another, while each subinterval was subdivided into the following time points: [50/2^14 ^50/2^12 ^50/2^10 ^50/2^8 ^50/2^6 ^50/2^4 ^50/2^2 ^25 50]. We restricted our state space to all combinations of (*P, P_m_*) such that *P *∈ [0,110] and *P_m _*∈ [0,30], guaranteeing our obtained PDF is closer than 99.99% to the exact solution of the CME, at all considered time points. Probability values are colour-coded between values of 0 and 25%, where the end value corresponds to 25% or above.

**Figure 6:**
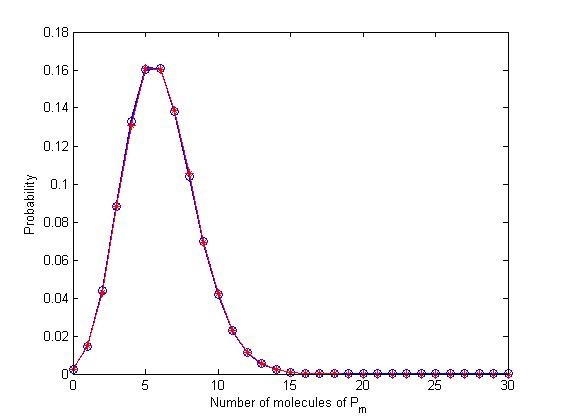
**PDF of adapted system and Poisson distribution**. The exact solution of the CME is shown at the endpoint of all input signal stimulation intervals (blue), as compared with 100,000 randomly generated samples of the Poisson distribution (red), where the parameter *λ *is equal to the first moment of the CME at any of those particular time points.

This brings us back to the arguments presented in the stochastic adaptation section. Namely, that adaptive behavior may not be inferred from single stochastic trajectories, since relaxation states revolving around the mean value do not necessarily represent the weighted majority in terms of probability. This can be readily observed from the values at the end states, represented as a heat map in Figure 5 implying large initial condition variations for the computation of each subsequent time interval. Actually, such wide variation can be analytically expected: it has been shown that a system composed of first order reactions that is both 'open' (i.e. including protein synthesis from source, hence violating conservation of mass) and has an initial Poisson distribution will remain a Poisson distribution at any time *t *> 0 [27]. Our results are entirely consistent with the theory (Figure 6). Hence, an adequate sample size and any other implications of the model have to be inferred from the underlying properties of this distribution (or else be thought as independent Binomial trials, the limit of which is the Poisson distribution).

Lastly, it should be noted there exist analytic results for 'open' and 'closed' systems of first order reactions, with arbitrary initial probability distributions. Hence, one can benefit from extracting an adequate sample size (or other properties) for arbitrary applications, provided all underlying reactions are unimolecular. In such cases, an appropriate PDF can be derived by the convolution of Poisson and Multinomial distributions, for any time *t *> 0 [27].

### Fold-change detection properties

Recent studies have highlighted the peculiar capability of certain systems to respond to fold-changes in the input signal, rather than to absolute differences in numbers of molecules [20,21]. This is the essence of Weber-Fechner's law, which states that the maximal response to a change in signal is inversely proportional to the background signal or, in other words, that the ratio of the smallest increment and the background intensity of a signal is constant. This can be better understood by quoting an analogy used in [33]: while it is easy to understand whispered voices (increment signal) inside a quiet room (background signal), it is very hard to notice someone shouting in our ear during a Rock concert.

The recently coined term fold-change detection (FCD) implies both Weber's law and perfect adaptation. However, simultaneous application of Weber's law and perfect adaptation do not necessarily yield FCD [20], and sufficient conditions have been presented to obtain it. Namely, if a system can be described by $\dot{x}\;=\;f(x,\;y,u)$ and $\dot{y}\;=\;g(x,y,u)$, where *y *corresponds to the output, *u *to the input signal, and *x *to the remaining chemical species, FCD can be achieved if the system is stable, shows perfect adaptation, and for *λ *> 0 it can be shown that *f*(*λx, y, λu*) = *λf*(*x, y, u*) and *g*(*λx, y, λu*) = *g*(*x, y, u*) [20].

It can be easily seen that the 'two-state protein' scheme does not satisfy these conditions, since:

$$\begin{array}{c} f\left(\lambda P,\;P_{m},\;\lambda A\right)\;=\;k_{s}\;+\;k_{r}P_{m}\;-\;\left(d_{P}+\lambda k_{a}A+k_{f}\right)\lambda P\\ \neq \;\lambda \left(k_{s}+k_{r}P_{m}\;-\;\left(d_{P}+k_{a}A+k_{f}\right)\right)\\ g\left(\lambda P,\;P_{m},\;\lambda A\right)\;=\;\left(k_{a}\lambda A+k_{f}\right)\lambda P\;-\;\left(k_{r}+d_{P_{m}}\right)P_{m}\\ \neq \;\left(k_{a}A+k_{f}\right)P\;-\;\left(k_{r}+d_{P_{m}}\right)P_{m}. \end{array}$$

Perhaps more intuitively, strict FCD properties could not be expected, since the 'two-state protein' scheme shows remarkable loss of sensitivity to repeated stimuli. Nevertheless, the criteria posed in [20] is only sufficient, and not necessary, so two questions are worth considering. First, to how many repetitions of the stimulus does FCD refer to? And second, to what extent are consecutive relaxations to a steady state expected to match?

**Figure 7:**
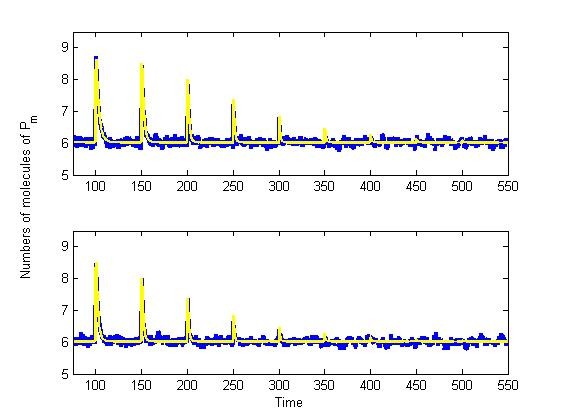
**Fold-change detection properties in two-state protein model**. Perfect adaptation two-state protein model with (a) A_1 _= [2^0 ^2^1 ^2^2 ^2^3 ^2^4 ^2^5 ^2^6 ^2^7 ^2^8 ^2^9 ^2^10^] and (b) A_2 _= 2*A_1_. Trajectories portray SSA simulations (blue), and solution of corresponding ODE system (yellow).

In the analysis published in [21], parameter variations in an incoherent feed-forward loop architecture are explored, and FCD is reported whenever the response to the two step stimuli is identical to within 10% in amplitude. By using this criteria in the 'two-state protein' scheme with fold-change input signal profiles A_1 _= [2^0 ^2^1 ^... 2^10^] and A_2 _= 2*A_1 _introduced at identical time points, FCD properties hold in two perspectives (Figure 7).

**Figure 8:**
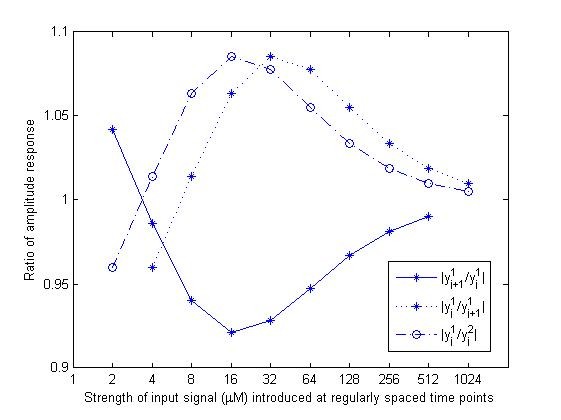
**Relative difference in amplitude response with fold-changes in input signal**. Perfect adaptation case with input profile A_1 _= [2^0 ^2^1 ^2^2 ^2^3 ^2^4 ^2^5 ^2^6 ^2^7 ^2^8 ^2^9 ^2^10^] *μM *and A_2 _= 2*A_1_. Relative differences were computed in three different ways: |*y*^1^_*j*+1_/*y*^1^_*j*_|, |*y*^1^_*j*_/*y*^1^_*j*+1_|, and |*y*^1^_*j*_/*y*^2^_*j*_|, where *y*^1 ^and *y*^2 ^are the ODE solutions using profiles A_1 _and A_2_, respectively, and index 'j' refers to the time point within interval 'j' where maximal response is attained.

**Figure 9:**
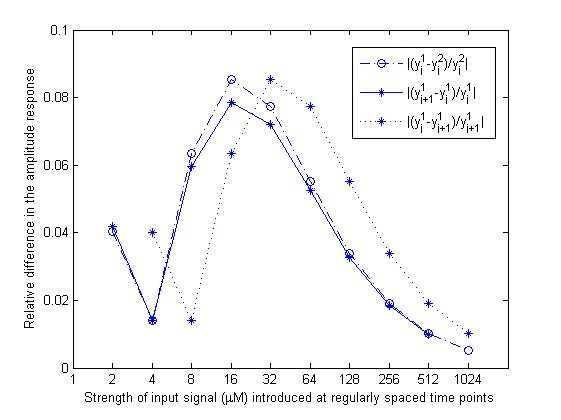
**Relative difference in amplitude response with fold-changes in input signal**. Perfect adaptation case with input profile A_1 _= [2^0 ^2^1 ^2^2 ^2^3 ^2^4 ^2^5 ^2^6 ^2^7 ^2^8 ^2^9 ^2^10^] *μM *and A_2 _= 2*A_1_. Relative differences were computed in three different ways: |(*y*^1^_*j*+1 _- *y*^1^_*j*_)/*y*^1^_*j*_|, |(*y*^1^_*j *_- *y*^1^_*j*+1_)/*y*^1^_*j*+1_|, and |(*y*^1^_*j *_- *y*^2^_*j*_)/*y*^2^_*j*_| where *y*^1 ^and *y*^2 ^are the ODE solutions using profiles A_1 _and A_2_, respectively, and index 'j' refers to the time point within interval 'j' where maximal response is attained. This analysis is equivalent to the criterion used in [21].

Both as an absolute ratio or by following the criteria used in [21], fold-stimulation yields responses within 10% of the amplitude not only for any two consecutive inputs, but also for a number of consecutive steps. So, even when the system looses sensitivity to the input signal, repeated stimulation preserves fold-change detection properties. Moreover, all responses in profile A_2 _are within 10% of the amplitude of those obtained with A_1 _(Figure 8 and 9). Most importantly, these results hold in the deterministic regime, and the first moment of the stochastic system.

**Figure 10:**
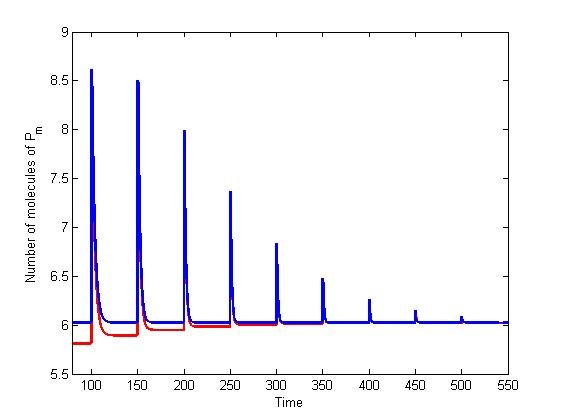
**Convergence of equilibrium values to the solution of a system with a degradation rate equal to zero**. Here we considered input profile A_1 _= [2^0 ^2^1 ^2^2 ^2^3 ^2^4 ^2^5 ^2^6 ^2^7 ^2^8 ^2^9 ^2^10^] *μM*. Solutions denote a system with *d_P _*= 0.01 (red) and *d_P _*= 0 (blue).

Lastly, in near-perfect adaptation systems, consecutive inputs yield slightly different steady states. With increasing numbers of input stimuli, the equilibrium values converge to the perfect adaptation case (Figure 10). This was readily observable from the equilibrium analysis (cf. Methods) as the input signal term would dominate, yielding convergence of the steady state solutions.

## Discussion

Our study of the 'two-state protein' in a stochastic setting lead us to simple yet illustrative examples on how biological ergodicity may be invalid, even in simple linear settings devoid of cell replication properties. A follow-up question could be: what would happen if the transitions between protein states were mediated by other molecular species in the system? In this case, one could consider discrete mediators (activators and deactivators, e.g. kinases and phosphatases) switching back and forth between the two protein states, as depicted in Figure 1B. Here, signal changes could refer to varying concentrations of the kinase and/or phosphatase.

**Figure 11:**
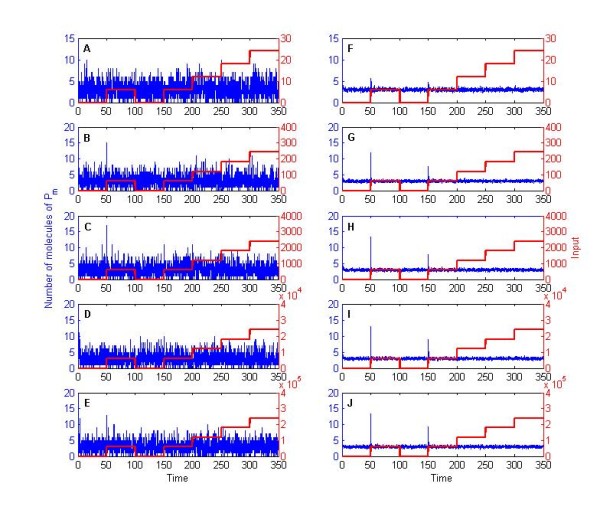
**Two-state protein model with kinases and phosphatases as discrete mediators**. (A-E) Single and (F-J) mean over a population of 50 cells, with initial concentration D(0) = 0.1 *μM*. Subfigures correspond to different levels of the signal A, in this case the kinase: (A, F) *F_i _*= 0.01, (B, G) *F_i _*= 0.1, (C, H) *F_i _*= 1, (D, I) *F_i _*= 10, and (E, J) *F_i _*= 100. Trajectories portray SSA simulations (blue), and input kinase signal (red). The following parameter values were used:$k_{s}=0.05,\;k_{f}=1,\;k_{r}=0.1,\;d_{P}=0.01,\;d_{P_{m}}=10,\;k_{a_{1}}=10^{3},\;k_{a_{2}}=k_{a_{3}}=k_{d_{1}}=k_{d_{2}}=k_{d_{3}}=10$.

**Figure 12:**
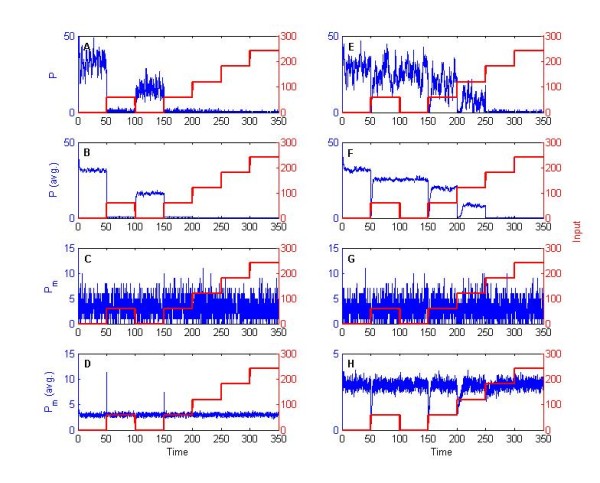
**Variations of key parameters in the two-state protein model, with kinases and phosphatases as discrete mediators**. Columns correspond to simulations using (A-D) parameter values of Figure 11; (E-H) *k*_*a*_2__= *k*_*a*_3__= 0.01. Rows correspond to single-cell time courses of (A, E) *P *and (C, G) *P_m_*; and the mean of a population of 50 cells of (B, F) *P *and (D, H) *P_m_*. Trajectories portray SSA simulations (blue), and input kinase signal (red).

Preliminary simulations show that all the properties studied for the simple two-state protein model can be achieved by this network topology. Such properties include perfect and near-perfect adaptation, as well as rupture of ergodicity, as exemplified by a varying kinase 'signal' in Figure 11. Qualitatively similar results can still be obtained by considering variations on the initial kinase and phosphatase concentrations (A(0), B(0) ∈ [0.01,0.1,1] *μM*) and single parameter values $\left(k_{a_{2}},\;k_{d_{1}},\;k_{d_{3}}\in [0.1,10,\;10^{3}]\right)$. The same holds for simultaneous variations of $\left(k_{d_{1}},\;k_{d_{3}}\right)$ or $\left(k_{a_{1}},\;k_{a_{2}}\right)$ while, interestingly, a completely different profile is obtained by simultaneous key variations of $\left(k_{a_{1}},\;k_{a_{2}},\;k_{a_{3}}\right)$. For instance, if we set these parameter values to (10^3^, 0.1, 0.1) a response that correlates negatively with changes in the signal is obtained (Figure 12). The latter can be explained by the high value of the ratio $k_{a_{1}}/k_{a_{2}}$, effectively 'trapping' *P *molecules in a bound configuration with the kinase, hence decreasing the numbers of molecules of both *P *and *P_m _*temporarily.

As can be intuitively expected, a similar effect in *P_m _*can be obtained by considering key variations of parameters $\left(k_{d_{1}},\;k_{d_{2}},\;k_{d_{3}}\right)$ with a varying phosphatase 'signal' (data not shown). Nonetheless, the profile of *P *will differ from the previous case, as the reaction set is not symmetric (Figure 1B), while it should be kept in mind a separation of timescales in the protein degradation rates was necessary to achieve adaptation. Additionally, changes in the kinase/phosphatase signal are not equivalent to the minimalistic 'two-state protein' system, since these discrete mediators are treated as molecular species and, as such, their numbers can fluctuate in time. Moreover, changes to the signal intensity only refer to unbound signal molecules and, in contrast to the linear case, the total number of signal molecules within the system can accumulate in time. The latter can be readily observed in distinct chemical scenarios (such as Figure 12) where a considerable number of kinase/phosphatase molecules can be 'trapped' in a bound configuration, the molecules of which remain within the system irrespective of changes in the signal.

In our kinase/phosphatase mediated adaptation example, obtaining an exact PDF solution of the CME would be computationally expensive, if not unfeasible, given the explosion in the number of reachable states. In this case a finite state projection using Krylov subspaces, such as [34] would be preferable. Alternatively, if the mediators are highly concentrated, one could 'decouple' them from the rest of the molecular species, yielding sets of unimolecular reactions, which in turn allows for obtaining an analytic closed solution. We will leave such analysis for a separate publication, given the high discrepancy of parameter values so far encountered and the significant deviation from the main focus of this work. However, considerations such as this, or deriving alternative closure of moments of the CME to account for non-linear terms, will greatly enhance future adaptation studies.

## Conclusions

In this paper, we have studied the effects of stochasticity in a 'two-state protein' scheme, providing an explanation of what adaptation means and entails in a stochastic setting. Namely, that an adaptation profile can be achieved by calculating the first moment of the CME, but that the underlying probability distribution might be wide enough to prevent one from making definite quick-and-dirty assertions going from a single cell to the population level, or the other way around.

In this sense, adaptation can many times be considered an emergent property of the collective system, restricting modelers/experimentalists to obtain large samples of time courses in order to infer properties of the system as a whole. The characteristics of such samples (e.g. a minimum number of single cells to depict population behavior), are necessarily described by the underlying probability distribution corresponding to the solution of the chemical master equation. Here, we have presented the overlap of an exact solution (the CME solved in matrix form), an analytical solution (the convolution of Poisson and Multinomial distributions, which is reduced to solely the Poisson distribution in our case), as well as trajectorial and deterministic solutions. Our analysis highlights the source of variability in single-cell scenarios, explaining the cause for rupture in ergodicity in a simple linear reaction network. We have also provided a clear perspective on how systems analysis with varying inputs can be addressed.

Additionally, we have shown how the minimalistic 'two- state protein' scheme displays fold-change detection properties in a stochastic setting. The latter refers to consecutive stimulation inputs and independent stimulations, and extends FCD properties to a system with near-perfect adaptation. Moreover, repeated stimulation preserved fold-change detection properties, despite loss of sensitivity to the input signal.

Lastly, we discussed extensions of the 'two-state protein' scheme by the consideration of discrete mediators (e.g. kinases and phosphatases). Our preliminary simulations show how such extensions retain many of the properties observed in the purely linear system, including loss of ergodicity.

To the best of our knowledge, this is the first time biological ergodicity has been shown to break down in a minimalistic linear architecture. The latter had been readily observed when cell replication rates are not homogeneous, or depend on the cell state, but never before in cell-cycle arrested scenarios composed of solely linear non-delayed terms. Furthermore, this is the first time fold-change detection properties have been studied in a stochastic setting.

## Methods

### Brief explanation of the Finite State Projection (FSP) method

For the purposes of this paper, the models are both bounded and finite, so we restrict our notation to N dimensions. If we define a vector ***p ***∈ ℝ*^n ^*such that each entry corresponds to the probability *P(x;t) *for each reachable state ***x***, we can think of its time evolution as $\dot{p}(t)\;=A\;p(t)$, where the matrix A = [a_ij_] contains the propensities and *a_jj _*= −Σ*_i≠j _a_ij_*, which basically means that each row of the matrix sums up to zero and the probability is conserved. Given an initial distribution ***p***(0), the solution at time t is ***p***(*t*) = exp(*t**A***) ***p***(0), where the matrix exponential is generally defined through its Taylor series expansion. If the reachable state space is large it may come in handy to consider a finite state projection [29], in which matrix ***A ***is replaced by ***A*_k_**, a k × k submatrix of the true operator ***A***, the corresponding indexed system states form the finite state projection and ***p***(*t_f_*) ≈ exp(*t_f_**A_k_***) ***p_k_***(0) is the approximation to ***p***(*t*) = exp(*t**A***) ***p***(0) at time *t_f_*. An approximation can be gradually improved by adding reachable states up to a pre-specified tolerance level.

**Figure 13:**
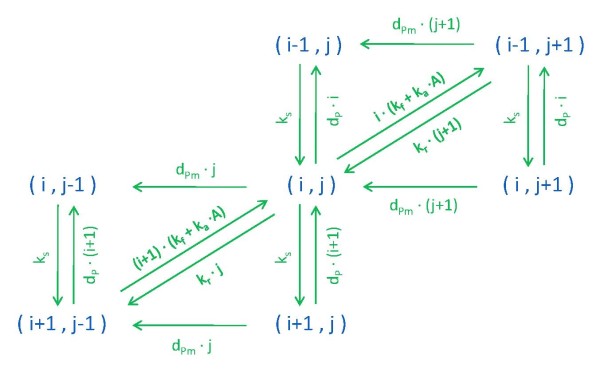
**Scheme used in finite state projections**.

In our case, the organization of the reachable states is shown in Figure 13 where (i, j) denotes the of molecules of (*P, P_m_*), and corresponds to the enumerated state of the system *j*(*M_P _*+ 1) + *i *+ 1. *M_P _*is the maximum number of molecules of *P *considered in the finite projection.

### Single cell vs. population measurements, from the simulation perspective

**Figure 14:**
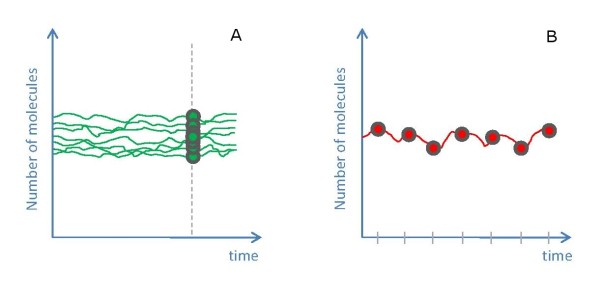
**Two different types of collecting sample points of stochastic simulations**. (A) Independent runs (corresponding to different cells) where for each simulation the number of molecules of P_m_ is collected at the same time, T*. (B) Single run (single cell experiment) where the number of molecules of P_m_ is collected at equally spaced time steps, all of which lie beyond T*. T* is assumed to be a point in time beyond the average time it takes for a system to reach its steady state.

In general, there are two ways in which stochasticity can be considered. The 'single cell' type or the 'multiple-cell' type (Figure 14). The first corresponds to studying possible states of the system in a single cell, by tracking molecular concentrations in time, once the system has reached 'equilibrium'. The second refers to comparison of multiple cells at one (or several) time point(s). In this study, preliminary stochastic simulations did not yield similar results for both types of analysis, the reason why we decided to report our results under both frameworks. In other applications, both frameworks yield similar simulation results as intrinsic stochastic noise is inherently a Markovian process and, by consequence, both cases can be considered mathematically equivalent. However, this is no longer the case in the two-state protein model presented here, which highlights how ergodicity can break down even in simple linear models.

### Solution methods and equilibrium values for the two-state protein model

The 'two-state' protein system (Figure 1) consists of six elementary reactions.

$$\begin{array}{c} \emptyset \overset{k_{s}}{\to }P\\ P\overset{k_{f}}{\to }P_{m}\;,P\overset{k_{a}\cdot A}{\to }P_{m}\;,\;P_{m}\overset{k_{r}}{\to }P\\ P\overset{d_{p}}{\to }\;\emptyset \;,\;P_{m}\;\overset{d_{p_{m}}}{\to }\;\emptyset \end{array}$$

In a deterministic setting, the time evolution of such a system can be described as:

$$\begin{array}{l} \frac{dP}{dt}\;=\;k_{s}+k_{r}P_{m}\;-\;(d_{P}\;+\;k_{f}\;+\;k_{a}A)\;P,\\ \frac{dP_{m}}{dt}\;=\;(k_{f}+k_{a}A)\;P\;-\;(k_{r}+d_{P_{m}})\;P_{m}. \end{array}$$

where *A *= *A*(*t*), and in this case *A*(*t*) is piecewise constant. The latter allowed us to use finite state projections in time intervals where *A*(*t*) = *c*, *c *∈ ℛ.

It can be easily seen that, at equilibrium

$$\begin{array}{l} P^{*}\;=\;\frac{k_{s}\;(k_{r}\;+\;d_{P_{m}})}{d_{P}\;k_{r}\;+\;d_{P_{m}}\;(d_{P}\;+\;k_{f}\;+\;k_{a}A)}\\ P_{m}^{*}\;=\;\frac{k_{s}\;(k_{f}\;+\;k_{a}A)}{d_{P}\;k_{r}\;+\;d_{P_{m}}\;(d_{P}\;+\;k_{f}\;+\;k_{a}A)} \end{array}$$

and if $d_{P_{m}}=0$, then P *= *k_s_*/*d_P _*and P_m _* = *k_s_*(*k_f _*+ *k_a_A*)/(*d_P_k_r_*). Similarly, if *d_P _*= 0 we have $P^{*}=k_{s}(k_{r}+d_{P_{m}})/d_{P_{m}}(k_{f}+k_{a}A)$ and $P_{m}^{*}=k_{s}/d_{P_{m}}$. As can be readily observed, when one of the degradation rates is equal to zero, the steady state solution of one molecular species is independent of the input signal.

In order to obtain transient solutions, we can formulate this as a matrix ODE problem. We shall consider here even more generalized problems, where *b *= *g*(*t*):

$$\begin{array}{l} \dot{P}=A(t)\;\cdot P+g(t),\;\text{where}\\ P={\left[P,P_{m}\right]}^{T},b={\left[k_{s},0\right]}^{T}\text{and}P(t_{0})=P_{0}. \end{array}$$

Once a fundamental solution φ(*t*) for the homogeneous problem $\left(\dot{P}=A(t)\;\cdot P,\;P(t_{0})=P_{0}\right)$ has been obtained, we can use the method of variation of parameters to construct a particular solution:

$$P(t)\;=\varphi (t)\varphi^{-1}(t_{0})P_{0}\;+\;\varphi (t)\;\int_{t_{0}}^{t}\varphi^{-1}(s)g(s)ds$$

As a stochastic differential equation (SDE) system with piecewise linear input signals, the 'two-state' protein system translates into:

$$\begin{array}{l} \left[\begin{array}{c} dP\\ dP_{m} \end{array}\right]=\;\left(\left[\begin{array}{c} k_{s}\\ 0 \end{array}\right]+\left[\begin{array}{cc} -d_{p}-k_{a}A-k_{f} & k_{r}\\ k_{a}A+k_{f} & -k_{r}-d_{p_{m}} \end{array}\right]\left[\begin{array}{c} dP\\ dP_{m} \end{array}\right]\right)dt\\ +\left[\begin{array}{cccccc} \sqrt{k_{s}} & -\sqrt{d_{p}P} & -\sqrt{k_{a}AP} & -\sqrt{k_{f}P} & \sqrt{k_{r}P_{m}} & 0\\ 0 & 0 & \sqrt{k_{a}AP} & \sqrt{k_{f}P} & -\sqrt{k_{r}P_{m}} & -\sqrt{d_{p_{m}}P_{m}} \end{array}\right]\;\left[\begin{array}{c} dW_{1}\\ dW_{2}\\ dW_{3}\\ dW_{4}\\ dW_{5}\\ dW_{6} \end{array}\right] \end{array}$$

and given the drift term is linear, the first moment of the SDE system will be identical to the ODE solution.

It is worth keeping in mind any system

$$dx(t)\;=\;(a_{1}(t)x(t)+a_{2}(t))dt\;+b(t)dW(t)$$

with a fundamental solution$\Phi_{\text{t},{\text{t}}_{0}}$ yields a transient solution prescribed by

$$\begin{array}{c} x(t)\;=\;\Phi_{\text{t},{\text{t}}_{0}}\left(x(t_{0})+\int_{t_{0}}^{t}a_{2}(s)\Phi_{\text{s},{\text{t}}_{0}}^{-1}ds\right)\\ +\Phi_{\text{t},{\text{t}}_{0}}\;\int_{t_{0}}^{t}b(s)\Phi_{\text{s},{\text{t}}_{0}}^{-1}\;dW_{s}. \end{array}$$

The fundamental solution will be

$$\Phi_{\text{t},{\text{t}}_{0}}=\exp(\int_{t_{0}}^{t}a_{1}(s)ds)$$

if matrix ***a*_1_**(*t*) commutes for all times *t*_1 _and *t*_2 _such that *t*_1 _≠ *t*_2 _. Otherwise, methods such as the Magnus expansion (or Fer, symmetric Fer, Cayley, etc.) can be used to obtain a fundamental solution.

## Authors' contributions

Both authors participated in designing the study. TML performed analysis of the CME and FSP simulations, AL performed SSA simulations. Both authors took part of the modeling, drafted, read, and approved the final manuscript.
